# Acute effects of different static stretching exercises orders on cardiovascular and autonomic responses

**DOI:** 10.1038/s41598-019-52055-2

**Published:** 2019-10-31

**Authors:** Gabriel Costa e Silva, Pablo B. Costa, Rodrigo Rodrigues da Conceição, Leonardo Pimenta, Roberto L. de Almeida, Monica A. Sato

**Affiliations:** 1 Department Morphology and Physiology, Faculdade de Medicina do ABC, Centro Universitario Saude ABC, Santo Andre, SP Brazil; 2 Research Group in Science of Human Movement, Colegio Pedro II, Rio de Janeiro, RJ Brazil; 3 Laboratory of Exercise Physiology and Human Performance of the Rural Federal University of Rio de Janeiro (LFDH/UFRRJ), Seropedica, RJ Brazil; 4 Department of Kinesiology, California State University, Fullerton, USA; 5 Department Endocrinology, Federal University of Sao Paulo (UNIFESP), São Paulo, SP Brazil

**Keywords:** Cardiology, Physiology

## Abstract

The present study examined the acute effects of static stretching (SS) exercise order on cardiac responses. Seventeen individuals were submitted to two experimental SS session: Order “A” (larger to small muscles groups) and Order “B” (small to larger muscles groups). Heart rate (HR), systolic (SBP) and diastolic blood pressure (DBP), rate-pressure product (RPP) oxygen saturation (SpO_2_), and heart rate variability (HRV) were measured at rest, midpoint of the session, immediately after the session, and in 5, 10, and 20 minutes after. SS increased HR and RPP in both orders, while reducing the rMSSD index and SpO_2_. In the order “A”, the SBP and DBP increased at the midpoint of the session. In the order “B”, the SBP and DBP increased only immediately after the end of the session. DBP and RPP significantly higher in order “A” compared to order “B” in the midpoint of the session. It was also demonstrated higher values of DBP and minor mean R-R intervals in order “B” at 10 min-post session. SS increased cardiac overload in both performed orders. The overload generated by the SS of the larger muscles groups was greater when compared to the smaller muscles groups, suggesting that the exercise order interferes in cardiac overload.

## Introduction

According to American College of Sport Medicine^1^, flexibility is one of the most important components of physical fitness related to health. Accordingly, static muscle stretching (SS) is an important exercise for maintaining and developing joint range of motion adequate to a healthy life. Without involving osteoarticular impact, the use of this exercise serves as an important tool in clinical prescription. Nevertheless, although widely used in the therapeutic environment, there is conflicting evidence on the use of SS exercises. Recent studies have demonstrated decreases in overall muscle-tendon stiffness after stretching^2–4^ and a decreases in muscle oxygen saturation (SpO_2_)^5^, decreases in force development^6–9^ and in lower recruitment of motor units^9,10^.

Konrad *et al*.^2^ demonstrated trough ultrasonography and dynamometry that 5 sets of 1 minute of SS reduce muscle stiffness and maximal isometric torque until 10 minutes after exercise. Fowles *et al*.^9^ reported that from after 60 seconds of SS support, muscle strength reduction can be observed, and Costa e Silva *et al*.^6^ reported only 30 seconds of SS can already reduce the levels of strength. Neural and structural mechanisms capable of generating such an adverse effect on performance seem to generate metabolic compensation due to a possible cardiovascular overload, according to the studies with important clinical contribution of Farinatti *et al*.^11^ and Lima *et al*.^12^, where the authors found higher cardiac and pressure responses as a function of SS.

Farinatti *et al*.^11^ examined 22 asymptomatic young men and cautioned that SS should be avoided in subjects with high risk for adverse cardiovascular events, because they observed significant increases in systolic blood pressure (SBP) and rate-pressure product (RPP) during the stretching sessions with and without the use of Valsalva maneuver. In addition, Farinatti *et al*.^11^ reported that muscle size may influence the magnitude of these cardiovascular responses. According to the authors^11^, SS of the large muscle groups seems to generate greater cardiac overload compared to the SS of the smaller groups. Despite the absence of studies on the topic, the results would become of great value for the choice of exercises and manipulation of the respective orders.

Exercise order is among the main methodological variables of a training program^1,12^. In agreement with ACSM^13^, this variable has great appeal in exercise prescriptions and has been researched by several investigations^14–26^. However, no study has examined the effects of the order of the muscles stretching exercises on the cardiovascular system. Currently, the ACSM^1^ has recommendations about the number of sets and stretch durations, nevertheless no information has been provided about the order of exercises.

Thus, the present study focused to investigate the acute effects of SS exercise order (larger to small muscle groups vs. small to larger muscle groups) on cardiovascular responses of heart rate (HR), blood pressure (BP), RPP, SpO_2_, and heart rate variability (HRV) in healthy individuals.

## Methods

### Participants

**Figure 1:**
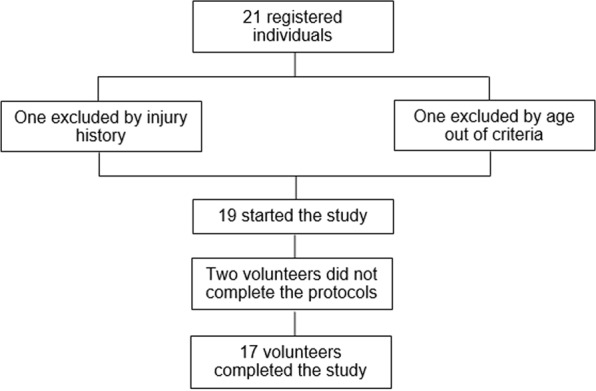
Volunteer Selection Flowchart.

Seventeen volunteers of both sexes (11 men and 6 women) were enrolled in the study (Table 1). The inclusion criteria were: age between 18 and 30 years, no history of injuries that limit joint range of motion, no hypo- or hypermobility, normotensive, familiarized with stretching exercises, no smokers and were advised for not drinking alcoholic beverages or caffeine in the 24 hours preceding the SS exercise bouts. The sample size was calculated using the G * Power 3.1 software. Based on an a priori analysis, we adopted a power of 0.80, α = 0.05, correlation coefficient of 0.5, correction nonsphericity of 1 and effect size of 0.26. This analysis of statistical power was performed to reduce the probability of type II error and to determine the minimum number of participants required for this investigation. We found that the sample size was sufficient to provide more than 81% statistical power. The research project was carried out in accordance to the recommendations of the National Health Council. We declare that all participants read and signed an informed consent form for study participation, and this study was approved by the Research Ethics Committee of the ABC Foundation School of Medicine (FMABC) (protocol number: 2.433.91) (Fig. 1).

**Table 1:** Descriptive data of the sample.

	Mass (Kg)	High (cm)	BMI (Kg/cm^2^)	Age (years)
**Men (n** = **11)**				
Mean ± SD	85.93 ± 13.36	179.53 ± 5.12	26.59 ± 3.23	26.09 ± 4.18
**Women (n** = **6)**				
Mean ± SD	60.16 ± 15.17	162.21 ± 7.41	22.69 ± 4.74	25.00 ± 4.33
**All (n** = **17)**				
Mean ± SD	78.84 ± 18.56	173.42 ± 10.31	25.21 ± 4.15	25.70 ± 4.13
CI 95% higher	67.30	168.10	23.07	23.58
CI 95% lower	86.39	178.70	27.35	27.83
SW p-value	0.9374	0.1204	0.5002	0.8517

BMI = Body Mass Index; SD = Standard Deviation; CI = Confidence Interval; SW = Shapiro-Wilk.

### Procedure

This study observed the cardiovascular and autonomic responses after two SS exercises orders based on the size of the stretched musculature. Participants were randomly submitted to two experimental stretching sessions with a minimum interval of 24 hours between sessions, named: Order “A” (from larger to small muscle groups): quadriceps, pectoralis, hamstrings, triceps, gastrocnemius, and biceps brachii. Order “B” (from small to larger muscle groups): biceps brachii, gastrocnemius, triceps, hamstring, pectoralis, and quadriceps. All volunteers participated in both experimental conditions. HR, SBP, diastolic blood pressure (DBP), SpO_2_, and HRV indexes were measured for 20 minutes at rest, at the midpoint of the session, immediately after the experimental session, and at 5, 10, and 20 minutes after the training session. The room temperature was approximately 22.06 ± 0.9 °C and relative air humidity was maintained at 60 ± 11.37%.

### Flexibility test and static stretching protocol

Before the beginning of the experimental procedures, volunteers underwent a test and retest (minimum of 24 h interval) of flexibility measured by the maximum amplitude reached passively by a goniometer (CARCI, Brazil). The following movements were measured: knee flexion (stretching of quadriceps), horizontal flexion of the shoulder (stretching of the pectoralis and biceps brachii), hip flexion (stretching of the hamstrings), elbow flexion (stretching of the triceps), and dorsiflexion (stretching of gastrocnemius). Three 3 attempts were carried out unilaterally, and the largest value obtained in the goniometer was considered. The positions evaluated were corresponding to the sustained positions during the SS exercises performed in the experimental protocols.

The SS was performed passively and unilaterally to the maximum point of discomfort through a scale of 0–10^5^. Three sets of 20 s were performed for each muscle group^1^, with a 20 s interval between sets and exercises. The SS for each muscle group was repeated using the maximum joint amplitude achieved and was measured with a goniometer. The breathing was guided with sound stimuli (2 seconds inspiration, 2 seconds expiration) during the entire session by the HRV Elite® software (Production Version 4.4.1, Asheville NC, USA).

### Heart rate and heart rate variability measurements

HRV Elite® software (Production Version 4.4.1, Asheville NC, USA) validated by Perrota *et al*.^27^ was used to measure HR and HRV variables. The electrode was moistened and positioned directly on the sternum (at the level of the xiphoid process), transferring the beats via Bluetooth to the software. Regarding HR, the last value shown on the device screen was recorded at each moment after each measurement was completed. In accordance with the procedures of the Task Force of the European Society of Cardiology and the North American Society of Pacing and Electrophysiology^28^, HRV analysis in the time domain was evaluated through the mean R-R intervals and square root of the mean squared differences of successive R-R intervals (rMSSD). The power signal intensity in the frequency range between 0.04–0.15 Hz (LF), and also in the frequency range between 0.15–0.40 Hz (HF), as well as the sympatho-vagal ratio (LF/HF) were used for HRV in the frequency domain.

### Blood pressure and rate- pressure product (RPP)

The measurement of SBP and DBP values was performed using an ambulatory blood pressure monitor (Boso-medicus, D-72417, Germany) using the oscillometric technique^29^, thus enabling automatic recording of blood pressure values. To obtain the RPP values, the HR values were multiplied by the SBP values (RPP = HR × SBP).

### Oxygen saturation

A finger pulse oximeter (digit Ox III N° AE-N2 Probasics, USA) was used to obtain oxygen saturation values. Finger pulse oximetry is considered an indirect measure of oxygen consumption^30^. To obtain the data, the probe was fixed on the index finger of the hand, which was supported on a fixed surface for stabilization.

### Statistical analysis

A Shapiro-Wilk test was used to verify the normality of the sample’s descriptive data. Reliability between test and retest sessions was analyzed using a two-factor random effect model intraclass correlation coefficient (ICC) and paired t-tests. A two-way ANOVA (repeated measures) was performed to examine differences in HR, SBP, DBP, RPP, SpO_2_, and HRV indexes after the experimental treatments (Order “A” vs. Order “B” and rest vs. midpoint of the session vs. end of the session vs. 5 min-after vs. 10 min-after vs. 20 min-after). Bonferroni post hoc test was then performed when appropriate. Statistical analyses were conducted using the statistical software package GraphPad Prism 5.0 and the significance level was set at p < 0.05.

## Results

The flexibility test and retest values presented excellent reproducibility (values between 0.90 and 0.99) and did not present a significant difference in the paired t test (p > 0.05).

### Heart rate

**Figure 2:**
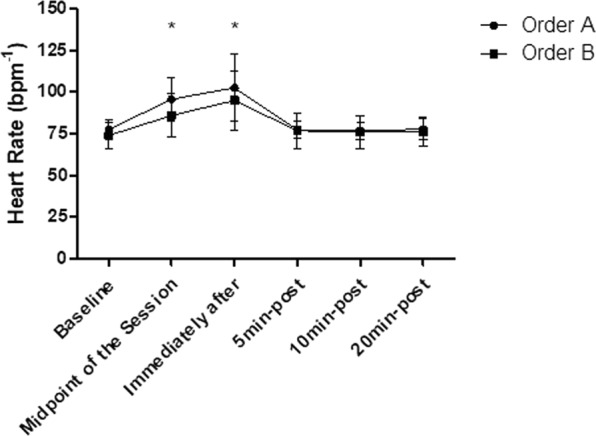
Mean and standard deviation of HR values in comparison between different exercise orders. *Represents significant difference from baseline.

The intra-group analysis showed that the order “A” and the order “B” both generated significant increases in the HR responses (p < 0.05), already observed in the midpoint of the session (p < 0.001) and persists increased immediately after the end of the session (p < 0.001). The HR has returned to baseline levels 5 min after stretching and remained in this range at 20 min after stretching in both groups (p > 0.05). In addition, according to the inter-group analysis, no difference was observed in HR between the order “A” and order “B” (p > 0.05), according to Fig. 2.

### Systolic blood pressure

**Figure 3:**
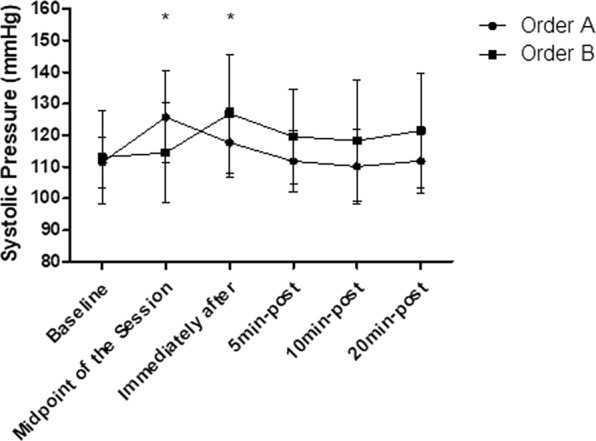
Mean and standard deviation of SBP values in comparison between different exercise orders. *Represents significant difference from the respective baseline.

The intra-group analysis showed that the order “A” and the order “B” both generated significant increases in the SBP responses (p < 0.05). In the order “A”, significant SBP increases were observed in the midpoint of the session (p < 0.01) and returned to baseline levels immediately after the end of the session, and remained in this range at 20 min after stretching (p > 0.05). In the order “B” significant SBP increases were observed immediately after the end of the session (p < 0.05) and returned to baseline levels 5 min after stretching and remained in this range at 20 min after stretching (p > 0.05). In addition, according to the inter-group analysis, no significant differences were observed between the order “A” and order “B” (p > 0.05), according to Fig. 3.

### Diastolic blood pressure

**Figure 4:**
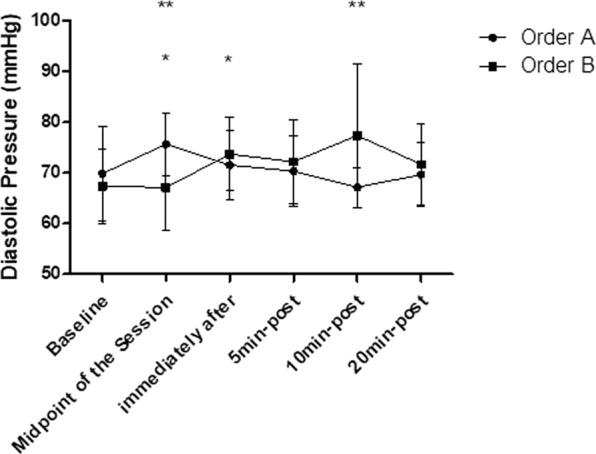
Mean and standard deviation of DBP values in comparison between different exercise orders. *Represents significant difference from the respective baseline. **Represents significant differences between different orders.

The intra-group analysis showed that the order “A” and the order “B” both generated significant increases in the DBP responses (p < 0.05). In the order “A”, significant increases in the DBP responses were observed in the midpoint of the session (p < 0.01), returned to baseline levels immediately after the end of the session and remained in this range at 20 min after stretching (p > 0.05). In relation to the analysis of the order “B”, we observed significant increases in the DBP responses immediately after the end of the session (p < 0.05), which remained increased (p < 0.05) until 20 min after stretching. In relation to the inter-group analysis, were found significant differences between order “A” and order “B” at the midpoint of the session (p < 0.05) and in 10 min-after SS (p < 0.01), according to Fig. 4.

### Rate-Pressure product

**Figure 5:**
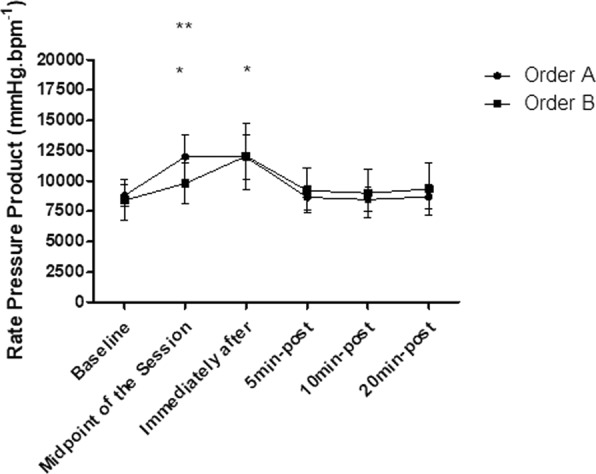
Mean and standard deviation of RPP values in comparison between different exercise orders. *Represents significant difference from the respective baseline. **Represents significant differences between different orders.

The intra-group analysis showed that the order “A” and the order “B” both generated significant increases in the RPP responses (p < 0.05), already observed in the midpoint of the session (p < 0.05) and persists increased immediately after the end of the session (p < 0.001). The RPP has returned to baseline levels 5 min after stretching and remained in this range at 20 min after stretching in both groups (p > 0.05). In addition, according to the inter-group analysis, were found significant differences between order “A” and order “B” at the midpoint of the session (p < 0.01), according to Fig. 5.

### Oxygen saturation

**Figure 6:**
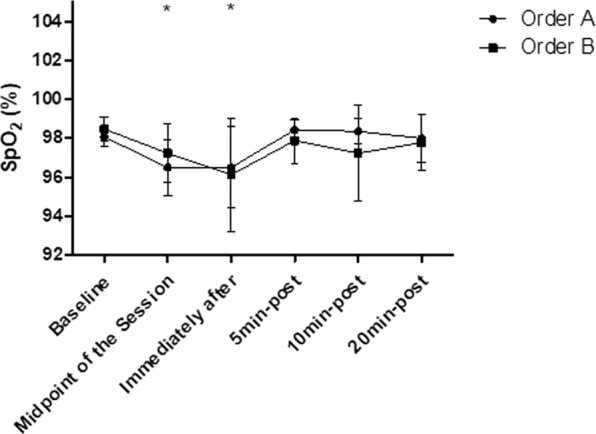
Mean and standard deviation of SpO_2_ values in comparison between different exercise orders. *Represents significant difference from the respective baseline.

The intra-group analysis showed that the order “A” and the order “B” both generated significant decreases in the SpO_2_ responses (p < 0.05), already observed in the midpoint of the session (p < 0.01) and persists decreased immediately after the end of the session (p < 0.01). The SpO_2_ has returned to baseline levels 5 min after stretching and remained in this range at 20 min after stretching in both groups (p > 0.05). In addition, according to the inter-group analysis, no significant differences were observed between the order “A” and order “B” (p > 0.05), according to Fig. 6.

### rMSSD

**Figure 7:**
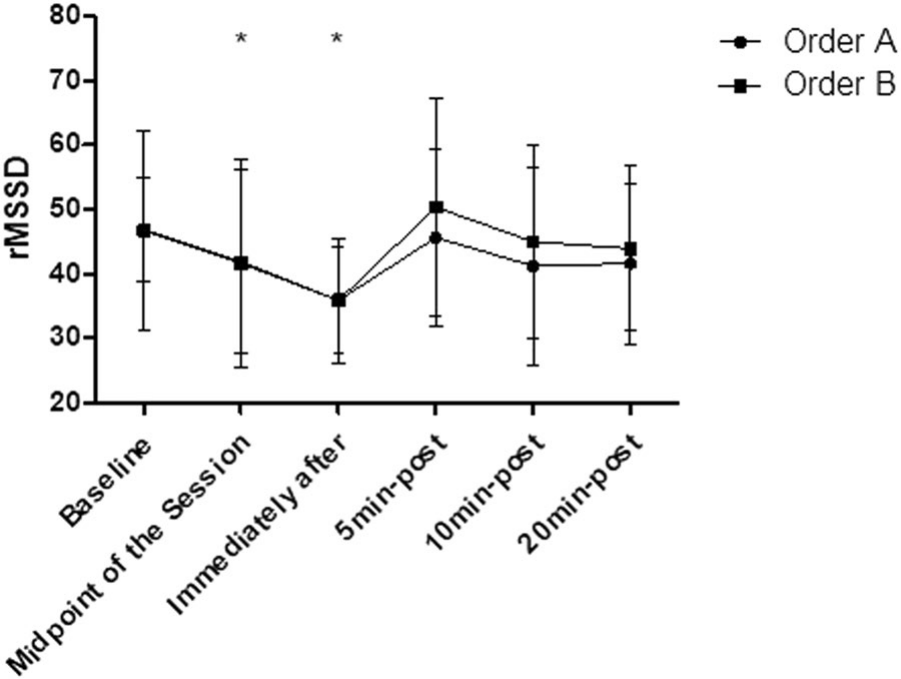
Mean and standard deviation of RMSSD values in comparison between different exercise orders. *Represents significant difference from the respective baseline.

The intra-group analysis showed that the order “A” and the order “B” both generated significant decreases (p < 0.05) in the rMSSD responses, already observed in the midpoint of the session (p < 0.01) and persists decreased immediately after the end of the session (p < 0.05). The rMSSD has returned to baseline levels 5 min after stretching and remained in this range at 20 min after stretching in both groups (p > 0.05). In addition, according to the inter-group analysis, no significant differences were observed between the order “A” and order “B” (p > 0.05), according to Fig. 7.

### LF

**Figure 8:**
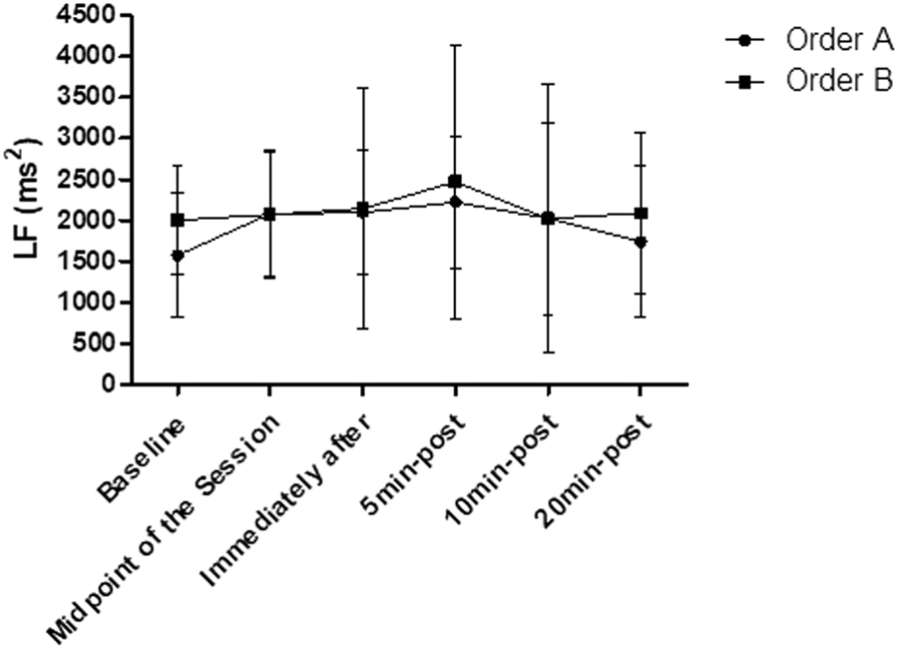
Mean and standard deviation of LF values in comparison between different exercise orders.

There are no significance in relation to the intra-group and inter-group LF analysis (p > 0.05), according to Fig. 8.

### HF

**Figure 9:**
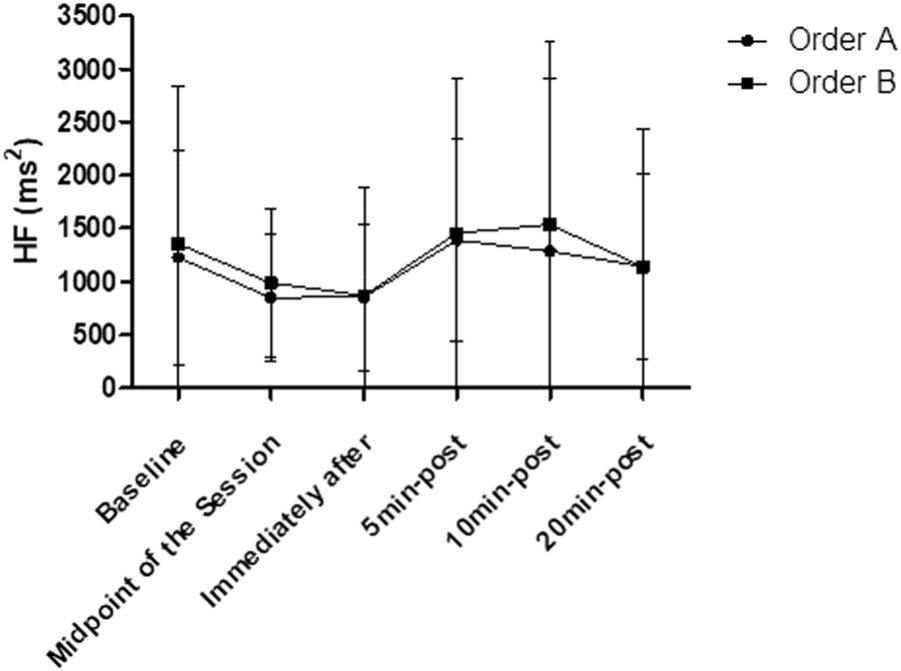
Mean and standard deviation of HF values in comparison between different exercise orders.

There are no significance in relation to the intra-group and inter-group HF analysis (p > 0.05), according to Fig. 9.

### LF/HF ratio

**Figure 10:**
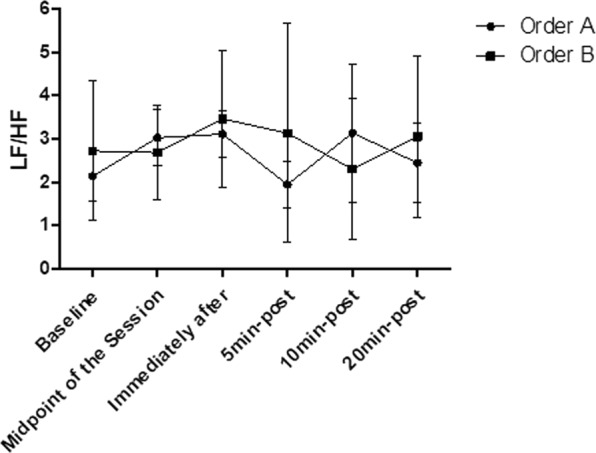
Mean and standard deviation of LF/HF values in comparison between different exercise orders.

There are no significance in relation to the intra-group and inter-group LF/HF ratio analysis (p > 0.05), according to Fig. 10.

### Mean R-R intervals

**Figure 11:**
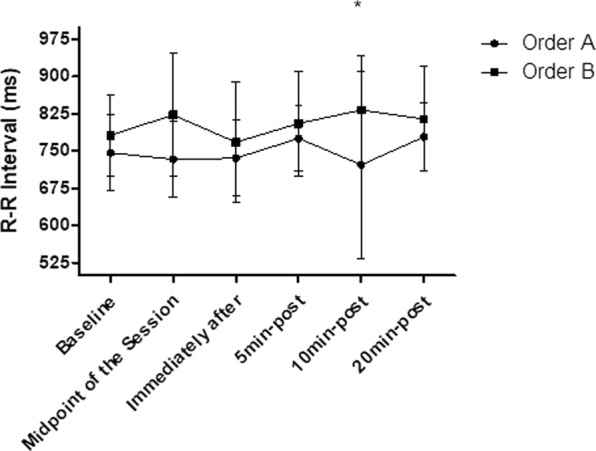
Mean and standard deviation of mean R-R intervals values comparing different orders of exercise. *Represents significant differences between different exercise orders.

There are no significant results in relation to the intra-group mean R-R intervals analysis in both orders (p > 0.05). However, in relation to the inter-group analysis were found significant differences between order “A” and order “B” at 10 min-after SS (p < 0.05), according to Fig. 11.

## Discussion

Through intra-group analysis, our best results demonstrated the SS session increase cardiac overload in both performed exercise order. Although inter-group results did not show significant differences immediately after the session in general, the data pointed to a higher cardiac overload in the SS of the larger muscle groups compared to the smaller muscle groups, suggesting that the exercise order interferes in cardiac overload. In the order “A”, SBP and DBP significantly increased at the midpoint of the session. Nevertheless, in the order “B”, the SBP and DBP significantly increased only immediately after the end of the session Accordingly, although the SS generated cardiac overload during and immediately after the experimental sessions, it was observed that even when performed up to the maximum joint amplitude, the values tend to return rapidly to the baseline levels within five minutes.

The present results demonstrated that SS does not allow the cardiovascular system to be overloaded for an extended period of time. Empirical knowledge and clinical prescriptions have usually recommended the accomplishment of stretching. Nevertheless, our findings showed that SS may increase heart overload and thereby, it should be prescribed with caution for individuals with a high cardiovascular risk. Additionally, in the present study the SS was performed to the maximum point of discomfort and probably the activation of peripheral pain pathways can enhance the HR and BP responses due to an increase in sympathetic modulation^31^.

Corroborating the findings of Farinatti *et al*.^11^, both orders of SS were able to significantly elevate HR, SBP, DBP, and RPP. In addition, our experiment significantly reduced the SpO_2_ and rMSSD values, thus suggesting a possible blood occlusion^5^, accompanied by a suppression of vagal modulation. Possibly such effects were due to the activation of mechanoceptors of small muscle fibers by SS exercises^32^. According to Mizuno *et al*.^33^, the pressor reflex mechanism demonstrated that exercises such as SS can generate alterations in autonomic nervous system (ANS), especially via the increase of the sympathetic modulation accompanied by vagal suppression regulated by medullary neurons.

Because of the muscular afferents, especially type III fibers, SS cause an increase in cardiac responses. In the present study, although they were not significantly different due to the high variability of the results, we observed that the SS showed a tendency to increase the LF and the LF/HF ratio, whereas the HF had a tendency to decrease. The increased cardiovascular responses observed in our findings are likely mediated by the reflex from the skeletal muscle (i.e., pressor reflex). Accordingly, the increases in this function are evoked by small muscle fibers afferents associated with the metaboreceptors, which are activated slowly during ischemic muscle contraction and by mechanoreceptors, which respond rapidly to muscular deformation evoked from SS. The afferent sensory information is then processed in the central nervous system, specifically within the nucleus of the solitary tract (NTS) that integrates the the sympatho-vagal balance pathways^34^.

Venturelli *et al*.^35^, in very recent study, investigated the sympatho-vagal relationship and blood circulation in 8 healthy young men submitted to repeated unilateral SS sets (5 × 45 s of knee flexion and 15 seconds of knee extension). The authors verified HR, cardiac output (CO), mean arterial pressure (MAP), HRV, BP variability and blood flow in rest and during repeated SS series. According to a computerized analysis of HRV the results showed that SS increased sympathetic modulation (≈20%) and decreased parasympathetic activity (≈30%) with a parasympathetic withdrawal prevalence, corroborating our results. From the device of photoplethysmography and ultrasonography, the authors observed that the HR, CO and MAP increased significantly during the SS sets and noticed that the blood flow was occluded. The Venturelli *et al*.^35^ data indicate that the high hemodynamic responses of the heart to SS were mainly influenced by the parasympathetic suppression.

The vagal rMSSD suppression could also be observed in the present study. Thus, SS exercises do not appear to promote cardiovascular protection, elevating HR, BP and suppressing local blood flow and the vagal drive to the heart. In the meantime, the present study demonstrated a return of the variables to the baseline levels five minutes after the end of the session, thus having a vagal increase in the post-exercise recovery period agreeing with Farinatti *et al*.^36^. Farinatti *et al*.^36^ submitted 10 men with reduced levels of flexibility to a SS session (3 repetitions of 30 seconds) involving trunk and hamstring musculature up to the maximum point of joint amplitude. The authors measured HR and HRV before, during and 30 minutes after the end of the session. After analysis in the time and frequency domains, the results demonstrated sympathetic activity rises during static stretching exercise and decreases at the end of the exercise, despite a rapid vagal return. These observations were confirmed in the present study through the values of HR, SBP, DBP, RPP, and the rMSSD index.

Lima *et al*.^12^ reported that SS exercises increase heart overload, elevating HR and RPP, which is consistent with our findings. In addition, previous studies^6,7,37^ demonstrated that pre-exercise SS causes impairment in the performance of activities requiring strength, and may decrease the supply of oxygen to the stretched muscle^5^, suggesting that negative effects can generate metabolic and autonomic compensations^38^. In addition, Farinatti *et al*.^11^, after submitting apparently healthy non-athletes to two experimental conditions (4 sets of 30 seconds vs. 4 sets of 30 seconds with the Valsalva Maneuver) reported that SS significantly increased SBP and RPP, with or without performing a Valsalva Maneuver. Corroborating the present results, the authors demonstrated larger muscle groups are capable of generating more expressive responses when compared to the smaller groups.

The heart rate variability may be influenced by several factors, for example the differences between sexes. A study of 83 subjects of both sexes found higher heart rate variability in women. According to the authors, this difference is due to the protective effect of estrogen^39^. In this sense, Dutra *et al*.^40^ pointed that men have an increased sympathetic response compared to women, while women have a vagal predominance. Even with this possible vagal increase in women, our results demonstrated that static stretching was able to significantly suppress parasympathetic drive in both exercise orders.

Nevertheless, AIbinet *et al*.^41^, after 12 weeks of intervention with physical exercise, found similar improvement in vagal modulation through the standard deviation of R-R intervals (SDNN), rMSSD and HF indexes in men and women. Nagashima *et al*.^42^ showed significant improvement in heart rate recovery, which indirectly reflects post-exercise parasympathetic reactivation, also in men and women. The authors included men and women in the analysis, as well as Sandercock *et al*.^43^ who also evaluated young men and women in a study that demonstrated differences in autonomic modulation in individuals with higher levels of physical activity.

In our study, the Shapiro-Wilk test showed normality of data when analyzing men and women together. The procedures were randomized with counterbalanced input, each individual being compared to him- or herself. In addition to the fact that only six women do not justify a different statistical analysis, we chose to evaluate men and women together. The same choice was done by other authors^2,3,9^ who investigated the acute effects of static muscle stretching.

Our study is the first to show the effects of different orders of SS in small and large muscles groups on cardiovascular and autonomic responses. Thus, further studies involving different orders of stretching are still required to understand the mechanisms involved in the SS in order to be safety prescribed and widely diffused in the clinical setting.

## Conclusions

The SS session increases cardiac overload in both performed orders, pointing to greater overload when we compare the largest muscle groups to the smaller muscle groups. Thus, the exercise order interferes in cardiac overload. It is possible to observe that even when the joint amplitude is maximal, values tend to return rapidly to baseline levels. However, such exercises should be prescribed with caution by individuals with a high cardiovascular risk when the goal is cardiac preservation, especially by increased HR, SBP, DBP, RPP, and vagal suppression accompanied by reduction of oxygen supply.
